# Successful Rescue Ventilation of an Infant With a Laryngeal Mask Airway After Accidental Extubation in the Prone Position During Open Calvarial Reconstruction of a Craniosynostosis: A Case Report

**DOI:** 10.7759/cureus.47064

**Published:** 2023-10-15

**Authors:** Androcles Lester, Matthew R Paluska, Ricardo Falcon, Timothy R Petersen, Anil K Shetty, Codruta Soneru

**Affiliations:** 1 Department of Anesthesiology and Critical Care, University of New Mexico School of Medicine, Albuquerque, USA; 2 Department of Anesthesiology, Rocky Vista University College of Osteopathic Medicine, Englewood, USA; 3 Office of Graduate Medical Education, University of New Mexico School of Medicine, Albuquerque, USA; 4 Department of Obstetrics & Gynecology, University of New Mexico School of Medicine, Albuquerque, USA; 5 Department of Surgery, University of New Mexico School of Medicine, Albuquerque, USA

**Keywords:** pediatrics, laryngeal mask airway, craniosynostosis surgery, open calvarial reconstruction, prone position ventilation, accidental extubation

## Abstract

In this case report, we present a critical situation during an open calvarial reconstruction involving an 11-month-old infant. The patient experienced accidental extubation, requiring immediate intervention while in the prone position. Approximately two hours post-incision, ventilation became increasingly difficult due to a significant leak detected in the system. On closer inspection, it was observed that both the rubber tourniquet responsible for securing the anesthesia circuit and the tape that held the endotracheal tube in place had become loosened.

In response to this emergency, the decision was made to remove the displaced endotracheal tube. We successfully introduced a 1.5 laryngeal mask airway (LMA; Unique™, Teleflex Incorporated, Wayne, PA), which restored ventilation. The patient maintained stable oxygen levels throughout this emergency period, displaying no signs of desaturation. An hour post-intervention, the surgical procedure was completed. The process of removing the LMA was uneventful without any complications.

In the setting of emergent airway management, especially for patients in the prone position during surgical procedures, accidental extubation presents a challenge for healthcare providers. This case highlights the importance of prompt decision-making and having alternative airway devices on hand, such as an LMA.

## Introduction

The accidental extubation of a patient in the prone position constitutes a crisis in emergency airway management. This is particularly true when any attempt to return the patient to the supine position to facilitate re-intubation poses an unacceptable risk. As the literature available to guide clinical decision-making in this situation is limited, we include our example with a small number of case reports demonstrating laryngeal mask airway (LMA) insertion in the prone position as a successful rescue technique.

This article was previously presented as a poster at the Society for Pediatric Anesthesia Conference on March 3, 2017. Written Health Insurance Portability and Accountability Act authorization has been obtained from the patient's parents for the publication of this case report. This manuscript adheres to the applicable Enhancing the Quality and Transparency of Health Research Network (EQUATOR) guidelines using the CARE (Case Report) checklist.

## Case presentation

An 11-month-old male patient, weighing 9.8 kg, presented with a clover leaf deformity of his right posterior skull. His pertinent medical and surgical history included hydrocephalus status post placement of a right posterior ventriculoperitoneal shunt. The surgery performed was the first of a planned three-stage complex open calvarial reconstruction. This involved posterior calvaria expansion using a bi-coronal approach.

**Figure 1:**
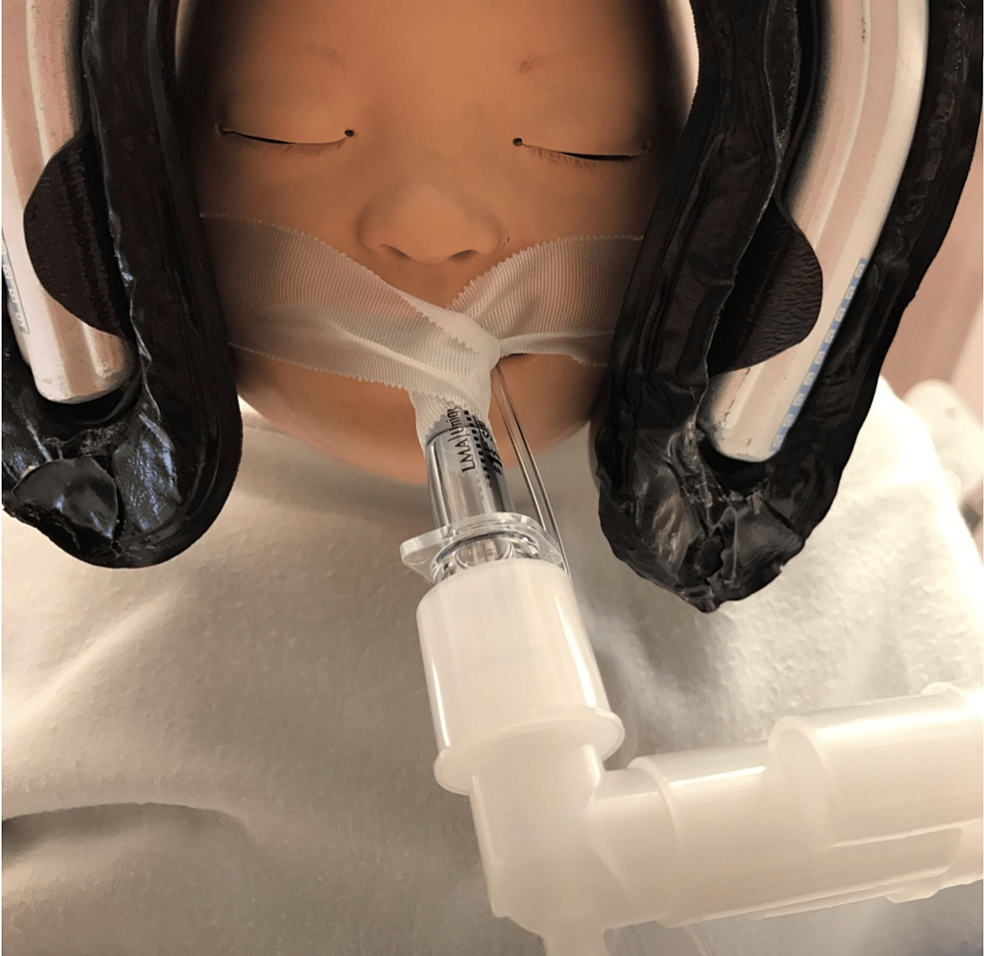
Reconstruction of the secured endotracheal tube with silk tape

General anesthesia was induced with 8% sevoflurane administered by mask, after which a 24-gauge intravenous (IV) catheter was established in the dorsum of the right foot. After receiving 10 pg IV fentanyl, 20 mg IV propofol, and 6 mg IV rocuronium, the patient was preoxygenated with 100% oxygen by bag-mask ventilation and placed in the sniffing position. Laryngoscopy was performed with a Miller 1 blade, which produced a Cormack-Lehane grade I view of the glottic orifice. The patient was intubated with a cuffed 4.0 endotracheal tube (ETT) placed using a stylet and secured at 12.5 cm, with an ETT leak at 20 cm H20. Successful ETT placement was confirmed through capnography, bilateral breath sounds by auscultation, and the observation of symmetric bilateral chest rise. The ETT was secured to the patient's face using silk tape (Figure 1).

Additional peripheral IV lines were established in the left hand and left foot as well as a 22-gauge radial arterial line in the right wrist. The patient was then positioned prone on gel rolls and a horseshoe headrest. The anesthesia circuit was additionally tethered to the OR table with a rubber tourniquet. The patient's head was shaved, the surgical field prepared and draped, and surgery commenced 93 minutes after intubation.

**Figure 2:**
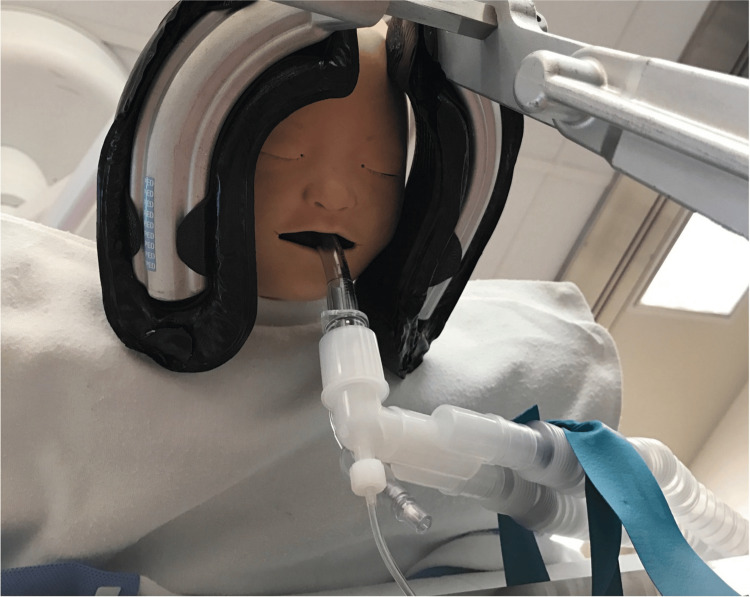
Reconstruction of the rescue laryngeal mask airway (Unique™) in place while the patient is in the prone position

The patient's face and ETT position were regularly monitored. Tidal volumes were optimal throughout the case. A significant leak was detected in the breathing circuit 135 minutes into the procedure, complicating ventilation. During the leak, the gas flow settings were maintained at 2 L/min for oxygen and 2 L/min for air. Auscultation revealed only faint breath sounds. Upon inspecting the ETT, it was found that the rubber tourniquet securing the anesthesia circuit to the operating table frame had loosened, placing the circuit's weight onto the tape. Further, the bloody irrigation fluid from the surgical field had saturated the silk tape, and it was detaching from the face. The ETT was displaced about 2 cm from its initial depth. The ETT was then removed and replaced with a 1.5 LMA (Unique™, Teleflex Incorporated, Wayne, PA) (seal <20 cm H_2_O) with the restoration of ventilation and no interval desaturation episodes (Figure 2). End-tidal CO_2_ measurements remained within normal limits after the restoration of ventilation.

The procedure continued for another 45 minutes without any further complications. The patient was then repositioned supine. Postoperative measures included reversal of neuromuscular blockade using 0.5 mg neostigmine and 0.1 mg glycopyrrolate, oral cavity suctioning, and LMA removal upon patient's return to consciousness and spontaneous breathing.

## Discussion

Accidental dislodgement of an ETT while in the prone position is a critical situation, especially in an infant with a limited physiologic reserve and, in this case, where an open calvaria prevents expedient return to a supine position. Placement of an LMA while prone, whether as a temporary solution or a replacement airway for the procedure's remainder, can be life-saving.

An ETT is often preferred for prone craniotomy cases such as this one due to its superior performance when hyperventilation is required to maintain reduced intracranial pressure, and it was our first choice. However, as the event transpired, we prioritized rapid restoration of ventilation, i.e., prompt restoration of overall ventilation was preferable to any investment of time and risk of patient movement to re-establish an ETT for its marginal benefits related to hyperventilation. The LMA choice offered a faster placement with reduced patient movement.

While prone, the jaw and tongue are displaced anteriorly by gravity to facilitate the passage of an LMA into the posterior oropharynx. The patient can be ventilated successfully if a proper seal is achieved [1].

Research on LMA placement in prone patients has primarily focused on elective situations, and consistent success has been reported. However, these studies rarely consider emergency scenarios [2]. Three case reports demonstrate success with an LMA as a rescue airway across a range of ages in pediatric patients. In one case, a neonate was successfully rescued with size 1 LMA approximately 20 minutes into a meningomyelocele repair after the ETT had slipped out [3]. Another case describes the successful placement of an LMA in a prone five-year-old female undergoing a decompressive craniectomy after the ETT was dislodged 127 minutes into the case [4]. Finally, an LMA was successfully placed for a 12-year-old female undergoing a spinal fusion for scoliosis after her ETT was accidentally removed [5].

Contrasting with the successful use of an LMA in these cases is a case of prone extubation reported by Thiel et al. [6]. In that case, the patient was a 62-year-old male, and the ETT dislodgement event occurred after most of the multiple-level cervical fusion had been performed. In that case, airway management was quite complex, and LMA replacement was more difficult, perhaps due to edema from extensive airway manipulation.

Difficulty placing an LMA and obtaining an adequate seal in the prone position can occur. It is likely secondary to mucosal edema in the oropharynx after a prolonged surgical course in the prone position and excessive administration of intravenous fluids [7]. Careful selection of an appropriately sized LMA with a high demonstrable leak pressure beforehand may optimize the chances of success of this maneuver [8,9].

Although the current evidence is insufficient for a definitive statement, our case and the three prior pediatric case reports suggest that an LMA can be successfully inserted for a prone patient. This can be a temporary measure when an ETT is dislodged until the patient can be reverted to the supine position and reintubated. Although accidental extubation events are infrequent, surgical plans for prone-positioned surgeries should be equipped to handle such airway emergencies.

## Conclusions

In the setting of emergent airway management, especially for patients in the prone position during surgical procedures, accidental extubation presents a challenge for healthcare providers. Our case emphasizes the potential life-saving efficacy of utilizing an LMA as a substitute or temporary airway in such situations, particularly in pediatric patients with limited physiologic reserves and in whom any head movement presents an unacceptable risk. The anatomical implications of the prone position often ease the placement of an LMA, provided that a sufficient seal can be obtained. While anecdotal evidence from case reports, including ours, highlights this technique's success, anesthesia teams must anticipate and be prepared for these rare emergencies. Our case further emphasizes the importance of careful monitoring and preventive measures, such as secure ETT taping and circuit anchoring, to minimize the risk of such events.
